# Reviewer acknowledgement

**DOI:** 10.1186/1756-3305-6-52

**Published:** 2013-03-18

**Authors:** Chris Arme

**Affiliations:** 1 Huxley Building, Keele University, ST5 5BG, UK

## Abstract

**Contributing reviewers:**

*Parasites & Vectors* would like to thank the following for their assistance with peer review of manuscripts for the journal in 2012.

## 

Elisabeth Aberer

Austria

Nicole Achee

United States of America

Alvaro Acosta-Serrano

United Kingdom

Ishag Adam

Sudan

Emily Adams

Netherlands

Abdulhamid Ahmed

Malaysia

Cihangir Akdemir

Turkey

Samer Alasaad

Spain

Abebe Alemu

Ethiopia

Sandra Allan

United States of America

Alberto Allepuz

Spain

Hesham Al-Mekhlafi

Malaysia

Bulent Alten

Turkey

Boran Altincicek

Germany

Cosme Alvarado-Esquivel

Mexico

Gema Alvarez Garcia

Spain

Carlos Roberto Alves

Brazil

Maria Lourdes Amarillo

Philippines

Ahmad Amro

Palestinian Territory

John Anderson

United States of America

Anges

Yadouleton Benin

Daniela Antolova

Slovakia

Christophe Antonio-Nkondjio

Cameroon

Victor H. Aquino

Brazil

Alex Asidi

United Kingdom

Stephen Attwood

China

Sarah Auburn

Australia

Samson Awolola

Nigeria

Ricardo Luiz Azevedo Pereira

Brazil

Leïla Bagny Beilhe

Cameroon

John Kevin Baird

Indonesia

Anna Bajer

Poland

Thomas Balenghien

France

Gad Baneth

Israel

Maria Dolores Bargues

Spain

Carolina Barillas-Mury

United States of America

Donald Barnard

United States of America

Matthew Baylis

United Kingdom

Melissa Beall

United States of America

Jerzy Behnke

United Kingdom

Andrea Bellelli

Italy

Glenn Bellis

Australia

Robert Bergquist

Sweden

Luigi Bertolotti

Italy

Martha Betson

United Kingdom

Frederic Beugnet

France

Quentin Bickle

United Kingdom

Jude Bigoga

Cameroon

Peter Billingsley

United States of America

Donal Bisanzio

United States of America

Damer Blake

United Kingdom

Brad Blitvich

United States of America

Raul Bobes

Mexico

Moses Bockarie

United Kingdom

Ken Boorom

United States of America

Art Borkent

Canada

Christian Bottomley

United Kingdom

Ali Bouattour

Tunisia

Sonia Boughattas

Tunisia

Catherine Bourgouin

France

Konstantina Boutsika

Switzerland

Dwight Bowman

United States of America

Janette Bradley

United Kingdom

Marieta Braks

Netherlands

Daniel Bray

United Kingdom

Reginaldo Brazil

Brazil

Corey Brelsfoard

United States of America

Emanuele Brianti

Italy

Olivier Johan Tavai Briët

Switzerland

Paul J. Brindley

United States of America

Basil Brooke

South Africa

Simon Brooker

United Kingdom

Daniel Brooks

Canada

Iris Bruchhaus

Germany

Fabrizio Bruschi

Italy

Christine Budke

United States of America

Syeda Tullu Bukhari

France

Stewart Burgess

United Kingdom

Guy Cabral

United States of America

Maurizio Calvitti

Italy

Cinzia Cantacessi

Australia

Van-Mai Cao-Lormeau

French Polynesia

Gioia Capelli

Italy

Luis Cardoso

Portugal

Rubén Marino Cardozo

Argentina

Dave D. Chadee

Trinidad and Tobago

Theeraphap Chareonviriyaphap

Thailand

Jian-Ping Chen

China

Cheng-Chen Chen

Taiwan

Zhaoguo Chen

China

Chee-Seng Chong

Singapore

Wej Choochote

Thailand

Edwin Claerebout

Belgium

Peter-Henning Clausen

Germany

Maureen Coetzee

South Africa

Anna Cohuet

France

Esther Collantes-Fernandez

Spain

David Cone

Canada

Jan Conn

United States of America

Vincent Corbel

France

Margarita Correa

Colombia

Jaime Costales

Ecuador

Gilles Cottrell

Benin

Jean Tenena Coulibaly

Cote d'Ivoire

Francis Cox

United Kingdom

Isra Cruz

Spain

Ary Gomes da Silva

Brazil

Roch Kounbobr Dabiré

Burkina Faso

Erik Daemon

Brazil

Milan Daniel

Czech Republic

Filipe Dantas-Torres

Brazil

Michael Dark

United States of America

Hans Dautel

Germany

Angharad Davies

United Kingdom

Tim Day

United States of America

Jose de la Fuente

United States of America

Claudio De Liberato

Italy

Stijn Deborggraeve

Belgium

Teun Dekker

Sweden

Jerome Depaquit

France

Marketa Derdakova

Slovakia

Ibrahima Dia

Senegal

Abdoulaye Diabate

United States of America

Diawo Diallo

Senegal

Amidou Diarra

Burkina Faso

Colette Dissous

France

Armel Djenontin

Benin

Gerhard Dobler

Germany

Michael J. Doenhoff

United Kingdom

Laura Dornak

United States of America

Pierre Dorny

Belgium

Simon Draper

United Kingdom

Michael Dryden

United States of America

Joseph D'Silva

United States of America

Aifang Du

China

Mike Duffy

Canada

Robert Duncan

United States of America

Remy Durand

France

Ravi Durvasula

United States of America

Patricia Dutra

Brazil

Gerard Duvallet

France

Naomi Dyer

United Kingdom

Rebecca Eisen

United States of America

Mark Eisler

United Kingdom

Hany Elsheikha

United Kingdom

Aidan Emery

United Kingdom

David Engman

United States of America

Agustín Estrada-Peña

Spain

Josiane Etang

Cameroon

Franco Falcone

United Kingdom

Chia-Kwung Fan

Taiwan

Chafika Faraj

Morocco

Marit Farenhorst

Netherlands

Robert Farkas

Hungary

Guido Favia

Italy

Andy Fenton

United Kingdom

Heather Ferguson

United Kingdom

Joana Ferreira Marques

Sweden

Gabor Foldvari

Hungary

Ian Kendrich Fontanilla

Philippines

Peter Foster

United Kingdom

Stenio Fragoso

Brazil

Antonio Frangipane di Regalbono

Italy

John Frean

South Africa

Mark Freeman

Malaysia

Michael French

United Kingdom

Qing Fu

China

Thomas Fürst

Switzerland

Xin Gao

United States of America

Amadou Garba

Niger

Tini Garske

United Kingdom

Robin Gasser

Australia

James Geary

United States of America

Timothy Geary

Canada

Peter Geldhof

Belgium

Claudio Genchi

Italy

David George

United Kingdom

Alec Gerry

United States of America

Peter Gething

United Kingdom

Annunziata Giangaspero

Italy

Alessio Giannelli

Italy

Wendy Gibson

United Kingdom

Carol Gilchrist

United States of America

John Gimnig

United States of America

Geoffrey Gimonneau

Burkina Faso

Andrew Githeko

Kenya

Raquel Gleiser

Argentina

Eva Gluenz

United Kingdom

Geoffrey Gobert

Australia

Maria Gomes

Brazil

Suzete Gomes

Brazil

David Gorla

Argentina

Roly Gosling

United States of America

Bruno Gottstein

Switzerland

Louis Clement Gouagna

Reunion

Nicodem James Govella

Tanzania

Daniel Grabner

Germany

Luigi Gradoni

Italy

Jeremy Gray

Ireland

Jamie Griffin

United Kingdom

Mario Grijalva

United States of America

Edmundo Grisard

Brazil

Felix Guerrero

United States of America

Ana Carolina Guimarães

Brazil

Monika Gulia-Nuss

United States of America

David Gurarie

United States of America

Martin Hall

United Kingdom

Omar Hamarsheh

Palestinian Territory

Sarah Hamer

United States of America

Afrim Hamidi

Serbia

Patrick Hamilton

United Kingdom

Haakon Hansen

Norway

Ralph Harbach

United Kingdom

Myriam Harry

France

Kathrin Hartelt

Germany

Moawia Hassan

Sudan

Frances Hawkes

United Kingdom

Polly Hayes

United Kingdom

Michelle Helinski

Uganda

Andrew Hemphill

Switzerland

Tineke Herremans

Netherlands

Geoff Hide

United Kingdom

Yousif Himeidan

Sudan

Hajime Hisaeda

Japan

Helene Hiwat

Suriname

Mary Hodges

Sierra Leone

Lindy Holden-Dye

United Kingdom

Nildimar Honório

Brazil

David Hoole

United Kingdom

Sándor Hornok

Hungary

Min Hu

China

Diana Huestis

United States of America

Hilary Hurd

United Kingdom

Ana Hurtado

Spain

Ivy Hurwitz

United States of America

Susan Imbahale

Kenya

Seth Irish

United Kingdom

Akira Ito

Japan

Jac J. Jacobs

Netherlands

Thomas G.T. Jaenson

Sweden

Wojciech Jarosz

Poland

Jean-Christophe Bambou

Guadeloupe

Jean-Michel Berenger

France

Ming-sen Jiang

China

Ryan Jochim

United States of America

Simon Jones

Canada

Christopher Jones

United Kingdom

Malcolm Jones

Australia

Bernhard Kaltenboeck

United States of America

Joseph Kamau

Kenya

Helge Kampen

Germany

Hirotaka Kanuka

Japan

Stefan Kanzok

United States of America

Christian Kaufmann

Switzerland

Shin-Ichiro Kawazu

Japan

Brian Kay

Australia

Mirdad Kazanji

Central African Republic

James Kazura

United States of America

Louise Kelly-Hope

United Kingdom

Malcolm Kennedy

United Kingdom

Gerry Killeen

Tanzania

Carsten Kirkeby

Denmark

Lucimar Kneipp

Brazil

Bart Knols

Netherlands

Dave Knox

United Kingdom

Dhanpat Kochar

India

Elena Kocianova

Slovakia

Alica Kocisova

Slovakia

Lizette Koekemoer

South Africa

Aneta Kostadinova

Bulgaria

Michail Kotsyfakis

Czech Republic

Guibehi Benjamin Koudou

United Kingdom

Michael Kristensen

Denmark

Jürgen Krücken

Germany

Andreas Krueger

Germany

Eliningaya Kweka

Tanzania

Oliver Kwok

United States of America

Marcelo Labruna

Brazil

Dusan Lalosevic

Serbia

Patrick Lammie

United States of America

Frederic Lardeux

Bolivia

Mauricio Lascano

United States of America

Scott Lawton

United Kingdom

Mengistu Legesse

Ethiopia

Francisco Lemos

Brazil

Song Liang

United States of America

Thomas Lindenstrøm

Denmark

Tim Littlewood

United Kingdom

James Logan

United Kingdom

Vincenzo Lorusso

United Kingdom

L. Philip Lounibos

United States of America

Asanterabi Lowassa

Tanzania

Shaohong Lu

China

Zhao-Rong Lun

China

Torleif Markussen Lunde

Norway

Vanessa Machault

France

Sutherland Maciver

United Kingdom

Charles Mackenzie

United States of America

Kenneth MacKenzie

United Kingdom

Claudio Mafra

Brazil

Rohela Mahmud

Malaysia

Marta Maia

United Kingdom

Viktoria Majlathova

Slovakia

Imna Malele

Tanzania

Richard Malik

Australia

Naiela Malik

United Kingdom

Sylvie Manguin

France

Tiago Miguel Lopes Martins

Portugal

Santiago Mas-Coma

Spain

Els Mathieu

United States of America

Keith Matthews

United Kingdom

Simonetta Mattiucci

Italy

Franz-Rainer Matuschka

Germany

Humphrey Mazigo

Tanzania

Charles Mbogo

Kenya

Paul McVeigh

United Kingdom

Hamish McWilliam

Australia

Jolyon Medlock

United Kingdom

Janet Meredith

United Kingdom

Wilfried Meyer

Germany

Isaura Meza

Mexico

Andrei Daniel Mihalca

Romania

Wolfgang Miller

Austria

Guadalupe Miro

Spain

Ladslaus Mnyone

Tanzania

Farrokh Modabber

Iran

Siti Nursheena Mohd Zain

Malaysia

Asif Mohmmed

India

Norhayati Moktar

Malaysia

Ricardo Molina

Spain

David Molyneux

United Kingdom

Antonio Montresor

Switzerland

Sarah Moore

United Kingdom

Jorge Moreno

Venezuela

Eric Morgan

United Kingdom

Liam Morrison

United Kingdom

Kate Mounsey

Australia

Adrian Mountford

United Kingdom

Devendra Mourya

India

Catherine Moyes

United Kingdom

Bradley Mullens

United States of America

Ingrid Muller

United Kingdom

Maria Anice Mureb Sallum

Brazil

Silvane Murta

Brazil

Ephantus J. Muturi

United States of America

Joseph Mwangangi

Kenya

Pauline Mwinzi

Kenya

Soraya Naem

Iran

Minoru Nakao

Japan

Simona Nardoni

Italy

Torsten Naucke

Germany

Raphael N'Guessan

Benin

Pin Nie

China

Alasdair Nisbet

United Kingdom

Sammy Njenga

Kenya

Ace North

United Kingdom

Nicholas Ogden

Canada

Nobuo Ohta

Japan

Oivind Oines

Norway

Gaetano Oliva

Italy

Annette Olsen

Denmark

Peter Olson

United Kingdom

Esther Orozco

Mexico

Michael Osae

Ghana

Mike Osta

Lebanon

Domenico Otranto

Italy

Yasushi Otsuka

Japan

Johnson Ouma

Kenya

Chris Oura

Trinidad and Tobago

Faye Ousmane

Senegal

Eunice Owino

Kenya

Richard Martin Oxborough

United Kingdom

Gil Padonou

Benin

Stephen Page

Australia

Clarisa B. Palatnik-de-Sousa

Brazil

Weiqing Pan

China

Rosario Panadero

Spain

Anna Papa

Greece

Paul Parham

United Kingdom

Susan Paskewitz

United States of America

Luiz Felipe Passero

Brazil

Christophe Paupy

Gabon

Ying-Hui Peng

China

Cedric Pennetier

Benin

Vera Lucia Pereira-Chioccola

Brazil

Eskild Petersen

Denmark

Kenneth Pfarr

Germany

Kurt Pfister

Germany

Joao Pinto

Portugal

Edward Platzer

United States of America

George Poinar

United States of America

Marco Pombi

Italy

Luiz Prestes-Carneiro

Brazil

Kristin Wear Prestrud

Norway

Concepcion Puerta

Colombia

Liu Quan

China

Rupert Quinnell

United Kingdom

Rafael Ramos

Italy

Reda Ramzy

Egypt

Sarah Randolph

United Kingdom

Hilary Ranson

United Kingdom

Paul Ready

United Kingdom

Lisa Reimer

Papua New Guinea

Frantisek Rettich

Czech Republic

Filiberto Reyes-Villanueva

Mexico

Jose Ribeiro

United States of America

Dania Richter

Germany

Aurélie Righetti

Switzerland

Laura Rinaldi

Italy

Annapaola Rizzoli

Italy

Lucy Robertson

Norway

Vicente Roca

Spain

David Roiz

Spain

David Rollinson

United Kingdom

Mahmoud Romeih

United States of America

Mara Rosenzvit

Argentina

Jim Rothwell

Australia

Ivo Rudolf

Czech Republic

Michael Rust

United States of America

Una Ryan

Australia

Amir Saeed

Sweden

Ma. Isabel Salazar

Mexico

Patrícia Salgueiro

Portugal

Oscar Salomon

Argentina

Richard Samuels

Brazil

Sammy Sam-Wobo

Nigeria

Christopher Sanders

United Kingdom

Pierre Sasal

French Polynesia

Yukita Sato

Japan

Somphou Sayasone

Laos

Vera Margarete Scarpassa

Brazil

Francis Schaffner

Switzerland

Thomas Schnieder

Germany

Manuela Schnyder

Switzerland

Gabriele Schoenian

Germany

Ernst-Jan Scholte

Netherlands

Ryan Schwarz

United States of America

Cristian Scutaru

Germany

Kate Searle

United Kingdom

David Severson

United States of America

Aklilu Seyoum

Zambia

Essam Shaalan

Egypt

Igor Sharakhov

United States of America

Amit Sharma

India

Jeffrey Shaw

Brazil

Chien-Ming Shih

Taiwan

Varda Shkap

Israel

Cornelia Silaghi

Germany

Marta Silva

United States of America

O.P. Singh

India

Neeloo Singh

India

Marianne Sinka

United Kingdom

Steven P. Sinkins

United Kingdom

Pavel Siroký

Czech Republic

Hannah Slater

United Kingdom

Peter Soboslay

Germany

Daniel Sojka

Czech Republic

Philippe Solano

France

Laia Solano-Gallego

Spain

Cesar Solorzano

United States of America

Marta Spakulova

Slovakia

Olivier Andre Ettore Sparagano

United Kingdom

Sabine Specht

Germany

Carolina Spiegel

Brazil

Belinda Lea Spillings

South Africa

Eva Spitalska

United Kingdom

Hein Sprong

Netherlands

Claire Standley

United States of America

Dorothee Stanneck

Germany

Michelle Stanton

United Kingdom

Peter Steinmann

Switzerland

Jan Sterba

Czech Republic

Dietmar Steverding

United Kingdom

Eileen Stillwaggon

United States of America

Patricia Hermes Stoco

Brazil

Steven Stoddard

United States of America

Russell Stothard

United Kingdom

Katja Strasek Smrdel

Slovenia

Yaowalark Sukthana

Thailand

Sinnathamby Surendran

Sri Lanka

Bernd Sures

Germany

Johari Surin

Malaysia

Vlasta Svobodova

Czech Republic

Emmanuel Swai

Tanzania

Arno Swart

Netherlands

Hiroshi Tachibana

Japan

Wesley Tack

Belgium

David Poumo Tchouassi

Kenya

Robert Tesh

United States of America

Saravanan Thangamani

United States of America

Stephanie Thomas

Germany

R. C. Andrew Thompson

Australia

Li-guang Tian

China

Ellen Tijsse-Klasen

Netherlands

Zhao Tongyan

China

Rebecca Traub

Australia

Donato Traversa

Italy

Frederic Tripet

United Kingdom

Wenbin Tuo

United States of America

Kevin Tyler

United Kingdom

Samuel Ugbomoiko

Nigeria

Shigehiko Uni

Malaysia

Juerg Utzinger

Switzerland

Glyn Vale

Zimbabwe

Gediminas Valkiunas

Lithuania

Denise Valle

Brazil

E.Tellervo Valtonen

Finland

Jerome Vanderberg

United States of America

Ana Vázquez González

Spain

Fernando E. Vega

United States of America

Bronislava Víchová

Slovakia

Elvina Viennet

Australia

Felipe Vigoder

Brazil

Petr Volf

Czech Republic

Georg von Samson-Himmelstjerna

Germany

John Vontas

Greece

Denis Voronin

United Kingdom

Indra Vythilingam

Malaysia

Yukiko Wagatsuma

Japan

Anthony Walker

United Kingdom

Julia Walochnik

Austria

Judd Walson

United States of America

Jun-Yun Wang

China

Samuel Wanji

Cameroon

Dorn Watthanakulpanich

Thailand

Mylene Weill

France

William Weir

United Kingdom

Susan Welburn

United Kingdom

Renata Welc-Faleciak

Poland

Michael White

United Kingdom

Caroline Wielinga

Australia

Stephen Wikel

United States of America

Thomas Wilke

Germany

Richard Wilkerson

United States of America

Barbara Willi

Switzerland

Anthony Wilson

United Kingdom

R Alan Wilson

United Kingdom

Mark Wilson

United Kingdom

Sonja Wolken

Germany

Tonia Woodberry

Australia

Patrick Woodburn

United Kingdom

Bo Wu

United States of America

Tetsuya Yanagida

Japan

Guojing Yang

China

Delenasaw Yewhalaw

Ethiopia

Qi Zheng

China

Xiao-Nong Zhou

China

Xiaohua Zhu

United States of America

Xing-Quan Zhu

China

Annetta Zintl

Ireland

Feng-Cai Zou

China

